# miR-135a Inhibits the Invasion of Cancer Cells via Suppression of ERRα

**DOI:** 10.1371/journal.pone.0156445

**Published:** 2016-05-26

**Authors:** Violaine Tribollet, Bruno Barenton, Auriane Kroiss, Séverine Vincent, Ling Zhang, Christelle Forcet, Catherine Cerutti, Séverine Périan, Nathalie Allioli, Jacques Samarut, Jean-Marc Vanacker

**Affiliations:** 1 Institut de Génomique Fonctionnelle de Lyon, Université de Lyon, Université Lyon 1, CNRS UMR5242, Ecole Normale Supérieure de Lyon, Lyon, France; 2 Institut des Sciences Pharmaceutiques et Biologiques, Faculté de Pharmacie, Université de Lyon, Lyon, France; 3 Faculté de Médecine Lyon-Sud, Université de Lyon, Lyon, France; 4 UMOMT, Centre Hospitalier Lyon-Sud, Hospices Civils de Lyon, Lyon, France; The Chinese University of Hong Kong, HONG KONG

## Abstract

MicroRNA-135a (miR-135a) down-modulates parameters of cancer progression and its expression is decreased in metastatic breast cancers (as compared to non-metastatic tumors) as well as in prostate tumors relative to normal tissue. These expression and activity patterns are opposite to those of the Estrogen-Related Receptor α (ERRα), an orphan member of the nuclear receptor family. Indeed high expression of ERRα correlates with poor prognosis in breast and prostate cancers, and the receptor promotes various traits of cancer aggressiveness including cell invasion. Here we show that miR-135a down-regulates the expression of ERRα through specific sequences of its 3’UTR. As a consequence miR-135a also reduces the expression of downstream targets of ERRα. miR-135a also decreases cell invasive potential in an ERRα-dependent manner. Our results suggest that the decreased expression of miR-135a in metastatic tumors leads to elevated ERRα expression, resulting in increased cell invasion capacities.

## Introduction

MicroRNAs (miRs) are small non-coding RNAs that, through a so-called “seed box” bind to specific complementary sequences in the 3’UTR of target mRNAs and induce their degradation or block the translation of the encoded protein [1]. Given the short size of the mRNA binding region in miRs and given that imperfect miR binding is in some cases sufficient to induce mRNA regulation, an individual miR can act on several mRNAs. This raises the possibility that an entire pathway/process may be controlled at several levels through a single miR. MiRs can promote or repress cancer initiation or progression, and deregulation of their expression is a common feature in these processes [2–5]. Human miR-135a is encoded by two genes localized on different chromosomes (3 and 12 for miR-135a1 and miR-135a2, respectively), producing an identical, active sequence. Contradictory results have been published concerning the effects of miR-135a on cancer progression. Reports indeed indicate that this microRNA could promote or repress various traits related to tumor aggressiveness such as proliferation, cell migration and invasion in colon, melanoma, breast or prostate cancer cell lines [6–12]. However it was shown that miR-135a expression is strongly decreased in metastatic breast tumors (compared to non-metastatic cancers, [10]) as well as in prostate tumors relative to normal tissue [11], suggesting that, at least in these tumor types, miR-135a counteracts cancer progression. In this respect, re-expression of miR-135a in non-expressing cells reduces their invasion potential. At least three molecular mechanisms have been proposed to account for this reduction. Depending on the study and on the cellular model, miR-135a has indeed been shown to decrease the expression of FAK, Runx2 and ROCK1/2, all regulations that lead to a reduction of cellular invasion [9–11].

The Estrogen-Related Receptor α (*ESRRA*, hereafter referred to as ERRα) is a member of the nuclear receptor superfamily. As such it displays a conserved centrally located DNA-binding domain as well as a C-terminally located putative ligand-binding domain that contacts co-activators and is necessary for transcriptional activation [13]. However no natural ligand of ERRα has been identified to date. ERRα is thus referred to as an “orphan” receptor and activates the transcription of its target genes in an apparent ligand-independent manner. ERRα regulates several physiopathological processes *in vivo* such as osteoblast differentiation and bone formation (reviewed in [14]), innate immunity [15–16] and energy metabolism (reviewed in [17–18]). In addition, high ERRα expression in cancers correlates with a poor prognosis in various cancer types ([19–25], reviewed in [26–28]). Consistently, ERRα has been shown to promote several traits of cancer progression, ranging from proliferation [29–30], epithelio-mesenchymal transition [31], resistance to hypoxia [32], angiogenesis [33], metabolic switch to aerobic glycolysis [34–35], cell migration and invasion [36–37]. ERRα increases cell invasion through at least two molecular mechanisms. On one hand, the receptor indeed activates Wnt11-dependent cascade, on another hand, ERRα indirectly fine-tunes RhoA stability to a level that is appropriate for orientated cell migration and invasion.

Here we show that re-introduction of miR-135a in non-expressing cells results in the down-regulation of ERRα expression at the mRNA and protein levels. This occurs through 3’UTR sequences that are complementary to miR-135a seed box. Consistently, the expression of ERRα targets genes is modulated by miR-135a in an ERRα-dependent manner. Furthermore, miR-135a over-expression decreases the invasion capacities of MDA-MB231 and PC3 cells (human mammary and prostate carcinoma cells, respectively). These capacities can be rescued by re-expression of an ERRα construct that is not targeted by miR-135a. Altogether, this points to ERRα as an alternative miR-135a target involved in the regulation of cell invasion.

## Materials and Methods

### Cells and Reagents

MDA-MB231 (human mammary epithelial cells) and PC3 (human prostate cells) were grown in Dulbecco’s modified Eagle’s medium (Invitrogen) supplemented with 10% fetal calf serum, 10 U/ml penicillin, 10 μg/ml streptomycin. LNCaP (human prostate cell) were grown in RPMI (Invitrogen) supplemented in the same manner. Cells were maintained in a humidified 5% CO2 atmosphere at 37°C. siRNAs and miRNAs were introduced into cells using Interferine (Ozyme) or Lipofectamine 2000 (Thermo Fisher), respectively, following the manufacturers’ instructions. ERRα siRNAs (sequences on S1 Table) were from Invitrogen (siERRα#1) and Dharmacon (siERRα#2). Pre-miRNAs (PM11126 for miR-135a; miR negative control n°2) were from Ambion.

### Expression Analysis

Total RNAs were extracted by guanidinium thiocyanate/phenol/chloroform and converted to first strand cDNA using IScript cDNA synthesis kit (Biorad). Real-time PCR were performed in a 96-well plate using the IQ SYBR Green Supermix (Biorad). Data were quantified by the ΔΔ-Ct method and normalized to RPLP0 (36b4) mRNA. Sequences of the primers used in this study are shown on S1 Table.

For western blot analysis, cells were lysed in RIPA buffer. Proteins (50 μg) were resolved on 10% SDS-PAGE, blotted onto PVDF (Millipore), probed with specific antibodies after saturation and revealed using an enhanced chemiluminescence detection system (ECL kit, Amersham Biosciences) with appropriate specific peroxydase conjugated Abs. Antibodies were from Gentex (ERRα, GTX108166), Enzo (hsp90, ADI-SPA-830) and Sigma (Flag, F-3165).

### Plasmids and Luciferase Assays

pSG5Flag-ΔA/B-ERRα has been described elsewhere [38]. Sequences on ESRRA (encoding ERRα) 3’UTR that are complementary to miR-135a seed box were predicted using TargetScan (http://www.targetscan.org), PicTar (http://pictar.mdc-berlin.de) and miRanda MiRBase (http://microrna.sanger.ac.uk) on-line available programs. A 400 bp fragment from the human ESRRA 3’UTR encompassing both predicted complementary sequences was cloned by PCR using MDA-MB231-originating retrotranscribed mRNA and primers flanked by XhoI and SalI restriction sites. Complementary sequences were mutated by recombinant PCR. After verification by sequencing, wild-type and mutated fragments were cloned in pmiRGLO dual expression vector (Promega) as XhoI-SalI inserts downstream of the firefly luciferase reporter sequence. Sequences of the oligonucleotides used for mutagenesis are available on S1 Table.

For transient transfections, 10^5^ MDA-MB231 cells were seeded in 24-well plates and transfected using Lipofectamine 2000, 50ng pmiRGLO plasmid and the indicated amount of pre-miR. Cells were lysed 48 h after transfection and reporter activities (renilla and firefly luciferases) were determined using standard methods.

### Invasion Assays

For invasion assays, cells (5. 10^4^) were suspended in 200μl DMEM/2% FCS and seeded on top of matrigel invasion chambers (Corning). Cells were allowed to migrate toward the lower chamber containing DMEM/10% FCS for 48 hrs. Matrigel was removed using cotton buds and cells were fixed 1 h with 4% formaldehyde, colored with 0.1% crystal violet and microphotographed. Invading cells were counted on whole well using Image J.

### Statistical Analysis

Statistical significance was determined using Student t-tests between groups.

## Results

### miR-135a Decreases ERRα Expression

**Fig 1:**
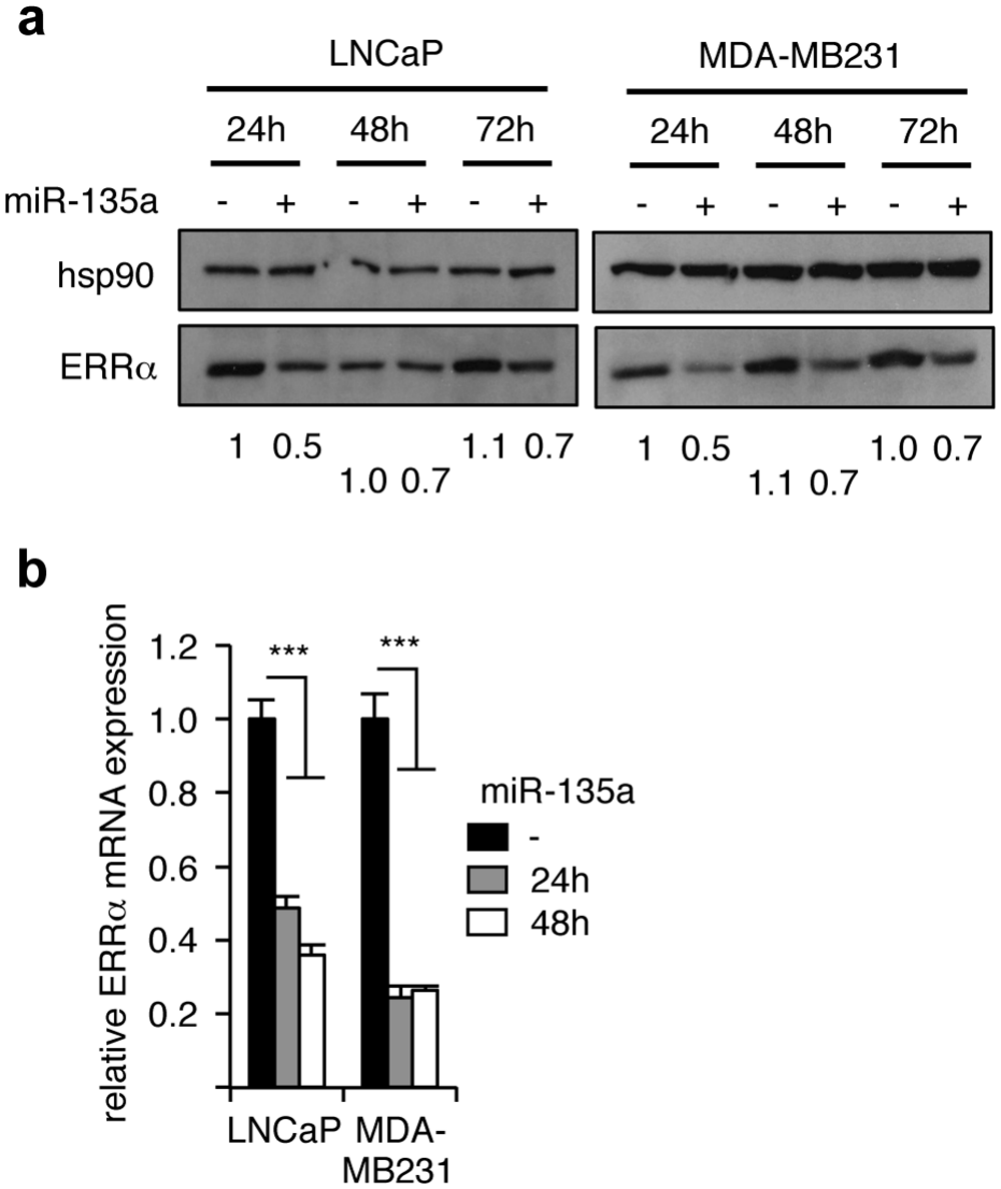
miR-135a reduces the expression of ERRα. The indicated cells were transfected with pre-miR-135a or pre-miR control (-) for the indicated time. **a.** Expression of the indicated proteins determined by western blot. Hsp90 was used as a loading control. Quantification of ERRα protein expression (displayed below the blots) is expressed relative that of hsp90, used as a loading control. **b.** Expression of ERRα corresponding mRNA analyzed by real-time PCR. Data are presented relative to pre-miR control samples and expressed relative to the expression of RPLP0. Shown is the average of experiments performed three times. Errors bars indicate SEM. ***: *p*<0.005.

During the course of a previous study, the consequences of pre-miR-135a over-expression in LNCaP human prostate cancer cells was analyzed in terms of cellular gene expression using a micro-array approach [11]. It was observed that the expression of the ERRα orphan nuclear receptor was decreased upon such a treatment. To validate these results, we transiently transfected pre-miR-135a in LNCaP cells and analyzed the expression of ERRα protein (Fig 1a). We found that the level of this receptor was reduced as soon as 24 h after transfection, an effect that persisted (albeit to a lesser extent) for 48 h. Similar results were also obtained in MDA-MB231 human mammary carcinoma cells. The ERRα-corresponding mRNA was also reduced 24h and 48h post-transfection in both LNCaP and MDA-MB231 cells, as judged by real-time PCR experiments (Fig 1b), suggesting a direct effect on mRNA.

**Fig 2:**
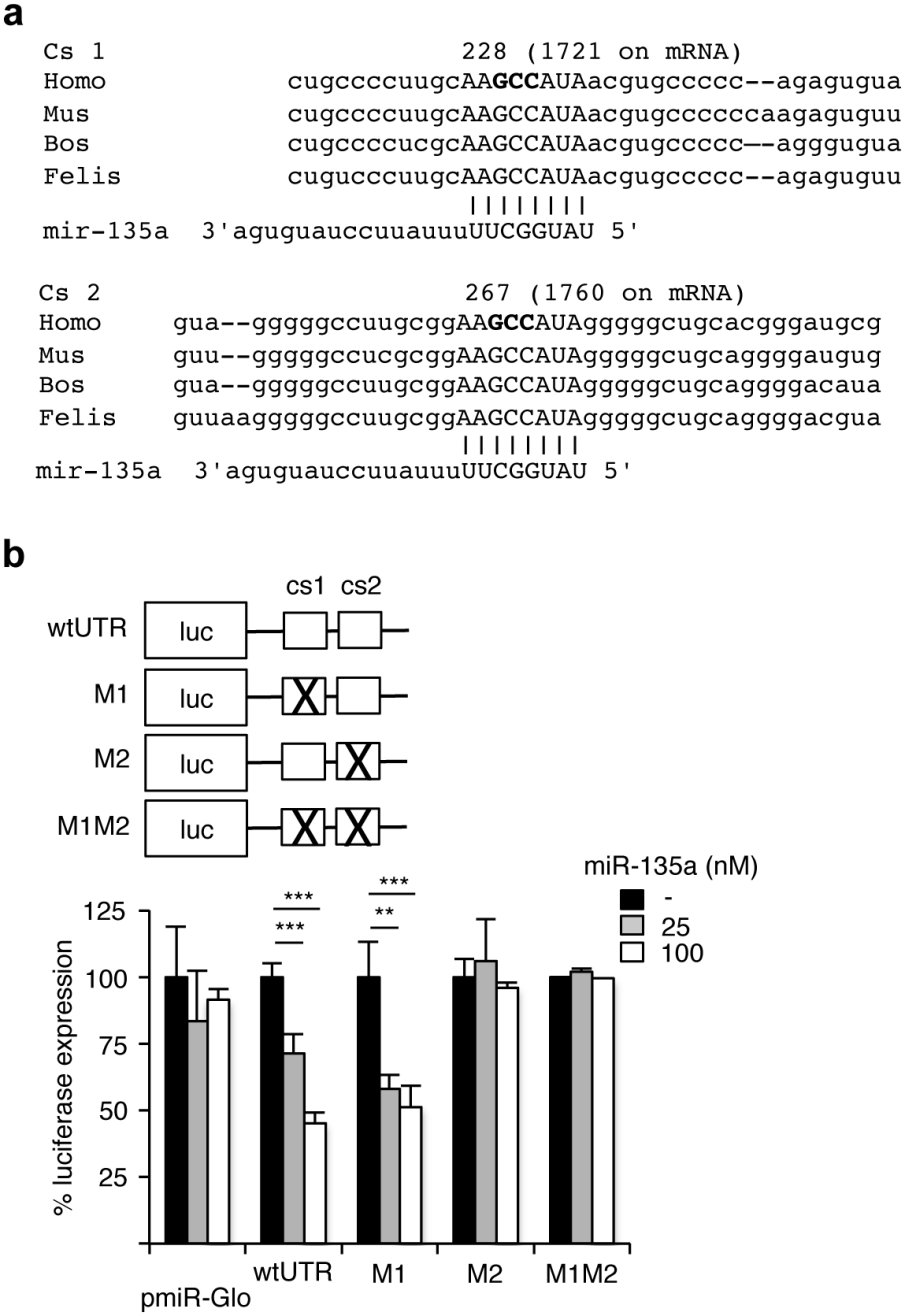
miR-135a acts on ERRα mRNA expression via its 3’UTR. **a.** Complementary sequences 1 and 2 (capital letters) on ERRα 3’UTR are displayed for the indicated species (*Homo sapiens*, *Mus musculus*, *Bos taurus* and *Felis silvestris*) and aligned to miR-135a sequence. Position of the first nucleotide of each complementary sequence (cs 1 and 2) is indicated relative the first 3’UTR nucleotide and relative to human mRNA start (under brackets). Nucleotides in bold letters were deleted in the corresponding mutants. **b.** Scheme of the generated mutants in pmiR-GLO is depicted at the top. MDA-MB231 cells were transiently transfected with the corresponding pmiR-GLO derivatives together with the indicated concentration of pre-miR-135a. Luciferase activities were determined 48 h after transfection and firefly luciferase activity was normalized to that of Renilla luciferase. Results are expressed for each plasmid as percent relative to transfection with pre-miR control and represent the mean of three independent experiments. Errors bars indicated SEM. **: *p*<0.01, ***: *p*<0.005.

We analyzed the human ERRα mRNA for microRNA recognition motives using three independent prediction programs (TargetScan, PicTar and miRanda), which yielded identical results. Two potential complementary sequences for miR-135a were identified in the 3’UTR with the highest (8-mer) binding probability (Fig 2a). Noteworthy the sequence as well as position of these two sites are highly conserved in mammals, but not in birds or amphibians, where no such corresponding sequence could be identified. To determine whether these complementary sequences are functional for miR-135a recognition, a 400 bp fragment of the 3’UTR (encompassing both potential sequences) was cloned downstream of Luciferase reporter gene into pmiRGLO plasmid. Each complementary sequence was then mutated alone or in combination. The resulting constructs were transfected in MDA-MB231 cells together with pre-miR-135a (Fig 2b). The wild type ERRα-derived fragment conferred miR-135a sensitivity, resulting in a 50% decrease in reporter activity. This effect was abolished upon mutation of complementary sequence 2, but not complementary sequence 1. This strongly suggests a direct effect of miR-135a on the 3’UTR of ERRα, in particular on complementary sequence 2, resulting in decreased levels of the corresponding mRNA and protein.

### miR-135a Regulates ERRα Molecular and Cellular Activities

**Fig 3:**
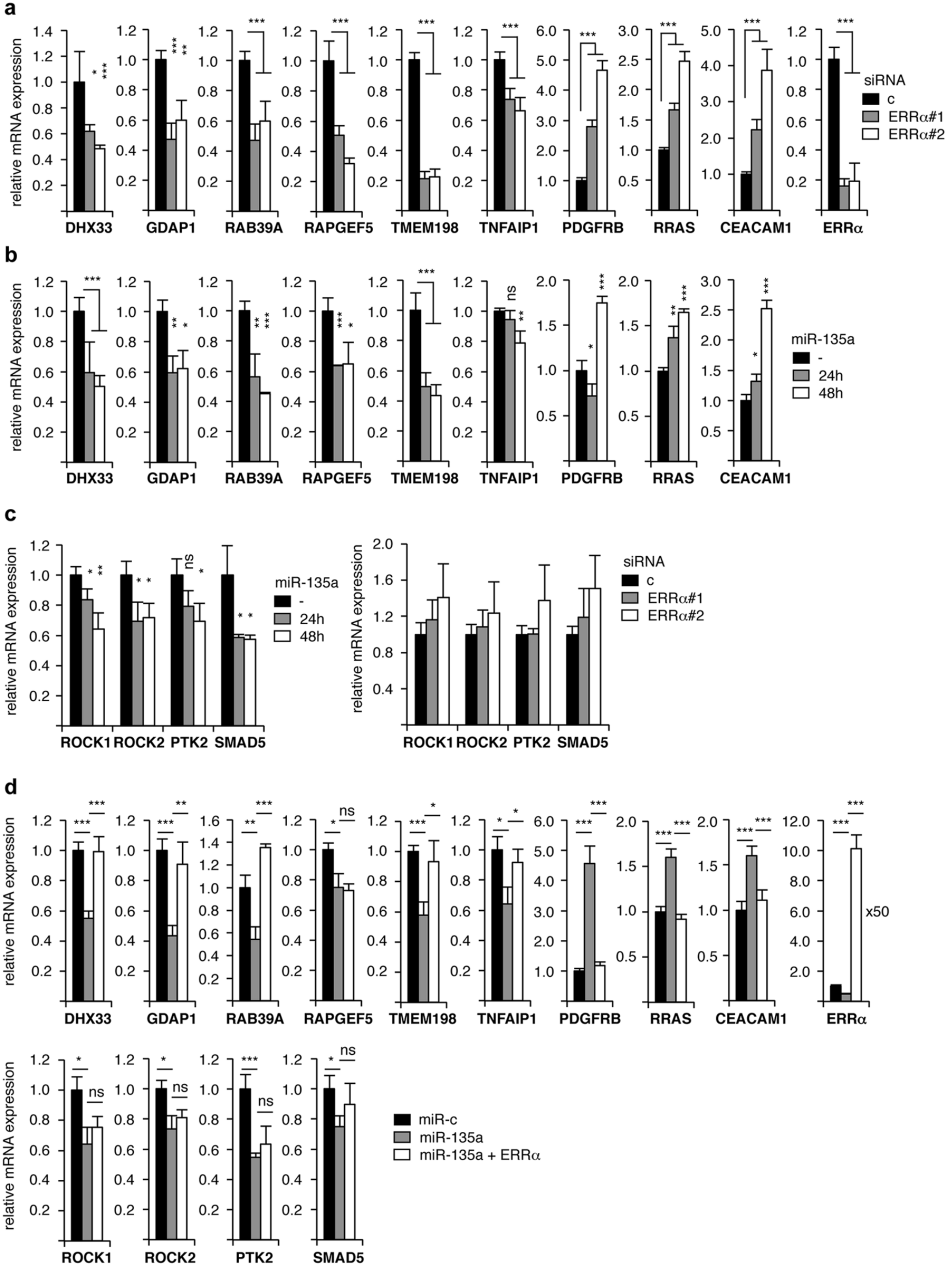
miR-135a regulates mRNA expression in ERRα-dependent and–independent manners. **a.** MDA-MB231 cells were transfected with the indicated siRNA (c: control siRNA) and RNA were extracted after 48 h. **b.** Cells were transfected with pre-miR-135a or control pre-miR (-). RNAs were extracted at the indicated time after transfection. Expression of ERRα targets was examined (**a**, **b**). **c.** Same experiment analyzing the expression of direct miR-135a target genes. **d.** Cells were transfected by pre-miR-135a and pSG5Flag empty vector (miR-135a) or ERRα-encoding plasmid (miR-135a + ERRα). As controls, cells were transfected by pre-miR control supplemented with pSG5Flag vector plasmid. RNAs were extracted after 48 h. Expression of the indicated genes was determined by real-time PCR. Data are presented relative to control and expressed relative to the expression of RPLP0. Shown is the average of three independent experiments in triplicate with error bars indicating SEM. *: *p*<0.05, **: *p*<0.01, ***: *p*<0.005, ns: non significant.

To analyze the consequences of this regulation, we examined whether the functions of ERRα can be impacted by miR-135a. ERRα acts as a transcription factor and its targets genes in MDA-MB231 cells have previously been identified through a transcriptomic RNA-sequencing approach [37]. We reasoned that miR-135a overexpression, leading to decreased ERRα levels, should also result in deregulation of the expression of these target genes. This hypothesis was examined on nine selected genes, which are, according to our transcriptomic approach, either positively (DHX33, GDAP1, RAB39A, RAPGEF5, TMEM198 and TNFAIP1) or negatively (PDGFRB, RRAS, CEACAM1) regulated by ERRα. Importantly, these genes were chosen since they were not predicted as direct miR-135a targets, according to TargetScan on-line program. Using two different siRNA against the receptor, we first verified that the expression of these genes is deregulated in the absence of ERRα (Fig 3a). We then analyzed the mRNA level of these ERRα targets upon miR-135a overexpression. As shown on Fig 3b, the expression of these genes was time-dependently regulated by transfection of pre-miR-135a. Of note, all miR-135a-induced deregulations were in the same direction as those induced by directly targeting ERRα. This further strengthens the hypothesis that the effect of miR-135a on these genes is ERRα-mediated. We also analyzed the expression of four genes (ROCK1, ROCK2, PTK2 and SMAD5) reported as miR-135a direct targets in the literature [10–11, 39]. As expected, the expression of these genes is time-dependently reduced upon pre-miR-135a overexpression (Fig 3c). However, none of these four genes appear deregulated in the absence of ERRα, as expected from our RNA-sequencing data (Fig 3c and [37]). We then used a rescue approach to question the possibility of an ERRα-dependent effect of miR-135a. To this end, cells were transfected by a miR-135a mimic supplemented or not by an ERRα construct that does not comprise its natural 3’UTR (*i*.*e*. without miR-135a complementary sequences). Consistently, deregulation of ERRα targets was rescued by re-introduction of the receptor (Fig 3d). In contrast, the expression of miR-135a direct targets (ROCK1, ROCK2, PTK2 and SMAD5), reduced upon overexpression of the pre-miR, was not restored by ERRα transfection. Together these data show that miR-135a impacts on gene expression in ERRα-dependent and–independent manners.

**Fig 4:**
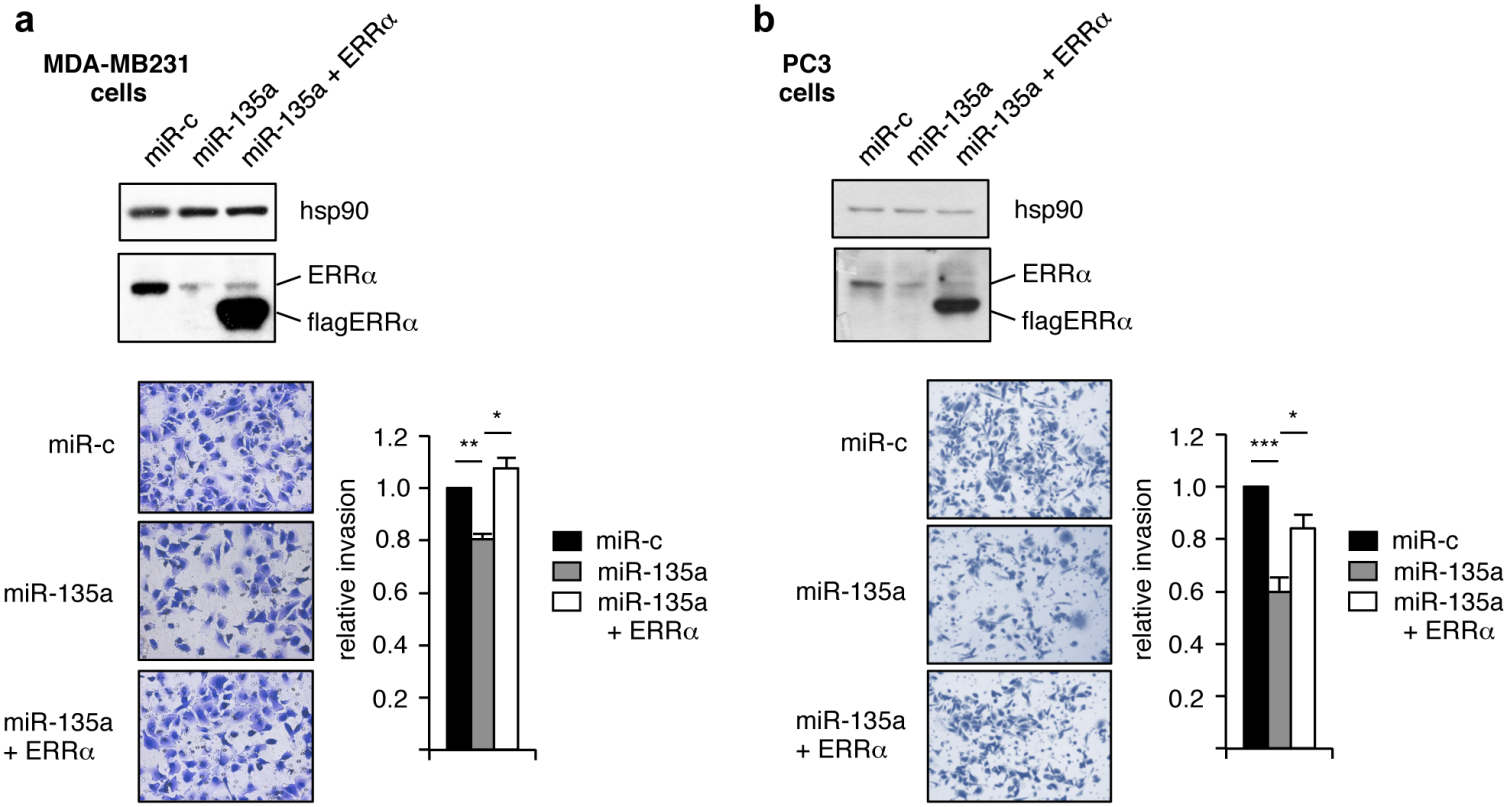
miR-135a decreases cell invasion by inhibiting ERRα expression. MDA-MB231 (**a**) or PC3 (**b**) cells were transfected with control miR (miR-c) or miR-135a mimic (both supplemented with empty pSGFlag plasmid) or miR-135a mimic supplemented with pSGFlag-ΔA/B-ERRα (deleted from its 3’UTR). Expression of the indicated proteins is shown in the upper panels. Cells were allowed to invade Matrigel on Boyden chamber assays. Invading cells were fixed and stained. Typical microphotographs are displayed on the left. Quantification was performed on whole well using Image J. Results are expressed relative to control miR conditions with error bars indicating sem. **: *p*<0.01, *: *p*<0.05, ns: non significant.

We next questioned whether functional effects of miR-135a could depend on ERRα. This receptor has been shown to be required for several traits related to cancer progression including cell migration and invasion [36–37]. On another hand miR-135a has been shown to reduce cell invasion [9, 11]. Using Boyden chamber invasion assays, we thus examined whether part of these latter activities can be mediated by ERRα inhibition. Overexpressing miR-135a mimic in MDA-MB231 cells led to a reduction of ERRα expression as well as to inhibition of cell invasion, as compared to controls (Fig 4a). In contrast, re-expressing a non-targetable ERRα construct fully restored cell invasion potential. Identical experiments conducted in the PC3 human prostate cell line yielded similar results (Fig 4b). We conclude that miR-135a can inhibit cell invasion, at least in part, through the inhibition of ERRα expression.

## Discussion

Discrepant results have been published concerning the role of miR-135a in cancer progression. Indeed, this microRNA has been reported to promote or inhibit traits of cancer aggressiveness [6–12]. Here we show that miR-135a reduces the expression of ERRα at the mRNA and protein levels, resulting in the modulation of the expression of the receptor’s target genes. Interestingly ERRα displays an expression pattern in cancers that is opposite to that of miR-135a. Indeed the receptor levels (mRNA and protein) are more elevated in tumors (relative to normal tissue) and its high expression correlates with a poor prognosis in various types of cancers such as those from the breast or the prostate (reviewed in [26–28]). This high expression could result from several mechanisms such as genomic amplification [40], a transcriptional autoregulatory loop [41], and the downregulation of microRNAs that target ERRα [25, 42]. Here we propose that a decrease in miR-135a expression may derepress that of the receptor. Consistent with its definition as a factor of poor prognosis, ERRα promotes several traits of cancer progression, such as EMT, angiogenesis, switch to aerobic glycolysis (Warburg effect) and resistance to hypoxia [31–35]. It is not known whether miR-135a is actually involved into counteracting the emergence of these processes. Nevertheless, miR-135a represses cell invasion [11], a parameter that is, in contrast, promoted by ERRα [36–37]. Our present results show that the inactivation of ERRα by miR-135a is instrumental in preventing cell invasion since this phenomenon can be restored by re-introduction of a non-targetable receptor. At least two non-mutually exclusive mechanisms have been evoked to account for the pro-invasive activities of the receptor. On the one hand, ERRα induces Wnt11 signaling which directly regulates cell invasion via an autoregulatory loop involving β-catenin [36]. On the other hand, the receptor positively regulates the expression of TNFAIP1, which reduces RhoA protein stability as well as the activity of its downstream effector ROCK1 [37]. In the absence of ERRα the increase in ROCK1 activity leads to an inhibition of cell invasion. Interestingly it was recently shown that miR-135a directly induces ROCK1 mRNA degradation, also resulting in inhibition of cell invasion [11]. In this respect, the reduction of miR-135a expression in cancers may result in uncontrolled ROCK1 expression that may become excessive and thereby inhibit cell invasion. However, the reduction of miR-135a also likely leads, in parallel, to increased ERRα expression. In turn the receptor reduces ROCK1 activity down to a level that is appropriate for cell invasion. In summary miR-135a and ERRα may form a multi-leveled regulatory network that fine-tunes ROCK1 activity.

Perusing the literature, it appears possible that this miR-135a- ERRα regulatory network may be effective in a completely different context. Indeed it has been shown that miR-135a directly down-regulates the expression of the master gene of osteoblast differentiation, Runx2, thereby preventing osteogenesis [39]. *Per se*, the observed BMP2-induced decrease in miR-135a expression may thus lead to uncontrolled Runx2 over-expression and thus premature osteoblast differentiation. On another hand, independent data have revealed that both Runx2 expression and activity are repressed by ERRα ([43–45], reviewed in [14]). The decrease in miR-135a expression may thus directly derepress the expression of both Runx2 and ERRα, the latter in turn moderating the activity of former. Such a network would eventually result in fine-tuning Runx2 activity, as described above for ROCK1. More work is required to demonstrate the accuracy of this hypothesis.

## Supporting Information

S1 Table Oligonucleotides used in this study. (PDF)
