# Supplementary material for: miR-135a Inhibits the Invasion of Cancer Cells via Suppression of ERRα
Source: PLoS One. 2016 May 26;11(5):e0156445. doi: 10.1371/journal.pone.0156445 (PMC4881992; doi:10.1371/journal.pone.0156445)
Supplement: S1 Table — (PDF) [file pone.0156445.s001.pdf]

Supplemental Table S1: Oligonucleotides used in this study

Expression studies in QPCR

	forward (5'-3')	reverse (5'-3')
RPLP0	GTCACTGTGCCAGCCCAGAA	TCAATGGTGCCCCTGGAGAT
ESRRA	CAAGCGCCTCTGCCTGGTCT	ACTCGATGCTCCCCTGGATG
DHX33	GCATCTGCTACCGGCTCTAC	GGTGAGCACATTTGGGACTT
ELK3	CCAAAGGCTTGGAAATCTCA	CGGAGTCAGAAGCAATCCAT
BSN	CCAGCCAAACTTCAACACCT	AGCCCTCTGCATCTGACAGT
GDAP1	AGCCTTGGTTTATGCGTTTG	CAGGCATTAACCTGGGTGTT
RAB39A	GTCGGCGTGGACTTCTTCT	TTGATCTGAACCGCTCCTGT
RAPGEF5	GCAGGAAGAGCATTGAGGAA	AACGCAGCTCCTCCTGTAA
TMEM198	GTGCTGTTTGTTTGGAGTCG	TCTCGGTAGCAGAGGAGGAA
TNFAIP1	CTCAGAACCGGCAAGAAATC	ATGTTGCACACAGGCTGGTA
ROCK1	CCTGATAACATGCTGCTGGA	CATCGTACCATGCCTTCCTT
ROCK2	TTTTGGTGAAGTGCAGTTGG	GCTATTGGCAAAGGCCATAA
PTK2	AGAAGGCCAATTTGGAGATG	CTCACGCTGTCCGAAGTACA
SMAD5	CAGCCCAACAACACTCCTTT	ATAGGCAGGAGGAGGCGTAT

Cloning and mutations

forward (5'-3')	reverse (5'-3')
cloning of ESRRA 3'UTR	
CTCGAGGCCACAGCCTGCTGGCAGGG	GTCGACGGAGTTATTGCTTCAAGGGG
mutation in seed box 1	
GCTGCCCCTTGCAAATAACGTGCCCCCAG	CTGGGGGCACGTTATTTGCAAGGGGCAGC
mutation in seed box 2	
GGGGCCTTGCGGAAATAGGGGGCTGCACG	CGTGCAGCCCCCTATTTCCGCAAGGCCCC

siRNAs

siERRα#1	
GGCAGAAACCUAUCUCAGGUU	CCUGAGAUAGGUUUCUGCCUC
siERRα#2	
GAAUGCACUGGUGUCACAUCUGCUG	CAGCAGAUGAGACACCAGUGCUUC
